# Altered hippocampal subfield volumes in major depressive disorder with and without anhedonia

**DOI:** 10.1186/s12888-023-05001-6

**Published:** 2023-07-25

**Authors:** Congchong Wu, Lili Jia, Qingli Mu, Zhe Fang, Hammza Jabbar Abdl Sattar Hamoudi, Manli Huang, Shaohua Hu, Peng Zhang, Yi Xu, Shaojia Lu

**Affiliations:** 1 Department of Psychiatry, The First Affiliated Hospital, Key Laboratory of Mental Disorder’s Management of Zhejiang Province, Zhejiang University School of Medicine, Zhejiang Engineering Center for Mathematical Mental Health, No. 79 Qingchun Road, Hangzhou, 310003 Zhejiang China; 2 Faculty of Clinical Medicine, Zhejiang University School of Medicine, Hangzhou, Zhejiang China; 3 Department of Clinical Psychology, The Fifth Peoples’ Hospital of Lin’an District, Hangzhou, Zhejiang China; 4 Department of Psychiatry, Affiliated Xiaoshan Hospital, Hangzhou Normal University, Hangzhou, 310003 Zhejiang China

**Keywords:** Major depressive disorder, Anhedonia, Hippocampus, Subfields

## Abstract

**Background:**

Previous neuroimaging findings have demonstrated the association between anhedonia and the hippocampus. However, few studies have focused on the structural changes in the hippocampus in major depressive disorder (MDD) patients with anhedonia. Meanwhile, considering that multiple and functionally specialized subfields of the hippocampus have their own signatures, the present study aimed to investigate the volumetric alterations of the hippocampus as well as its subfields in MDD patients with and without anhedonia.

**Methods:**

A total of 113 subjects, including 30 MDD patients with anhedonia, 40 MDD patients without anhedonia, and 43 healthy controls (HCs), were recruited in the study. All participants underwent high-resolution brain magnetic resonance imaging (MRI) scans, and the automated hippocampal substructure module in FreeSurfer 6.0 was used to evaluate the volumes of hippocampal subfields. We compared the volumetric differences in hippocampal subfields among the three groups by analysis of variance (ANOVA, *post hoc* Bonferroni), and partial correlation was used to explore the association between hippocampal subregion volumes and clinical characteristics.

**Results:**

ANOVA showed significant volumetric differences in the hippocampal subfields among the three groups in the left hippocampus head, mainly in the cornu ammonis (CA) 1, granule cell layer of the dentate gyrus (GC-ML-DG), and molecular layer (ML). Compared with HCs, both groups of MDD patients showed significantly smaller volumes in the whole left hippocampus head. Interestingly, further exploration revealed that only MDD patients with anhedonia had significantly reduced volumes in the left CA1, GC-ML-DG and ML when compared with HCs. No significant difference was found in the volumes of the hippocampal subfields between MDD patients without anhedonia and HCs, either the two groups of MDD patients. However, no association between hippocampal subfield volumes and clinical characteristics was found in either the subset of patients with anhedonia or in the patient group as a whole.

**Conclusions:**

These preliminary findings suggest that MDD patients with anhedonia exhibit unique atrophy of the hippocampus and that subfield abnormalities in the left CA1 and DG might be associated with anhedonia in MDD.

## Introduction

Major depressive disorder (MDD) is a seriously disabling psychiatric disorder worldwide with a high rate of morbidity and a significant burden of disease [1]. MDD is fraught with the challenges of heterogeneity of the clinical phenotype, that is, various biological mechanisms underlie the clinical presentations of MDD, which cause great difficulty in the treatment and prognosis of patients [2]. Recently, particular attention has been paid to the subdivision of different subtypes of depression, and the in-depth exploration of different symptoms contributes to the personalized management of patients with MDD [3].

Anhedonia is one of the two core symptoms of MDD, and MDD with anhedonia is regarded as a potential endophenotype of depression [4, 5]. Anhedonia is defined as a loss of interest or an inability to experience pleasure according to the fifth edition of the Diagnostic and Statistical Manual of Mental Disorders (DSM-5). Multiple studies have indicated that anhedonia is associated with poor prognosis [6], impaired social functioning [7], and increased risk of suicide and mortality [8] in MDD. Moreover, increasing evidence has showed that cognitive dysfunction and anhedonia are closely related and that anhedonic individuals exhibit more severe cognitive deficits, which contribute to psychosocial disabilities in patients with MDD [9–11]. Therefore, increasing attention has been given to the research on the anhedonic MDD subtype. Moreover, concentrating on the neural mechanism of anhedonia may promote a deeper understanding of the mechanisms in MDD.

Previous studies have revealed that anhedonia is related to deficits in the brain reward circuitry. Structural and functional changes in reward-related regions such as the ventral striatum, nucleus accumbens (NAcc), prefrontal cortex, amygdala, and hippocampus could induce the symptoms of anhedonia [12]. Among them, the hippocampus, which consists of the cornu ammonis (CA) 1 to 4, dentate gyrus (DG), and the subiculum, is an important structure for encoding and retrieving declarative memories [13], and it also plays an important role in the reward-related behaviors [14]. Numerous animal studies have demonstrated that the electrophysiological activity of hippocampal neurons is related to various aspects of reward, such as an increase in firing rates when approaching a reward site [15], and an increase in the rate of spike-wave ripples when gaining a reward [16], which promote the prediction and learning of reward locations [17, 18]. Moreover, reward-related behavioral deficits are linked to the decreased strength and impaired plasticity of hippocampal excitatory synapses [19].

In addition to preclinical evidence, clinical studies have also confirmed the role of the hippocampus in reward processing and indicated the association between anhedonia and hippocampal dysfunction. In healthy individuals, it was found that reward motivation could activate the hippocampus preceding memory formation in encoding tasks, which emphasized the effect of the hippocampus in reward anticipatory memory mechanisms [20]. Interestingly, an imaging study in patients with schizophrenia reported that hippocampal activity as well as NAcc was related to trait physical anhedonia during neutral word recognition [21]. Moreover, increased glucose metabolism in the hippocampus was found to be associated with reduced anhedonia in MDD [22]. These findings suggest that aberrant function of the hippocampus is involved in the development of anhedonia.

However, studies investigating the association between anhedonia and hippocampal volume changes in MDD are rare. Most recent studies have focused on patients with depressive episodes, while the findings are quite inconsistent. It was found that MDD patients showed decreased hippocampal volume in prior studies [23, 24]; however, some studies found no significant difference in hippocampal volume between patients with MDD and healthy individuals [25–27]. These conflicting conclusions might be attributed to different factors, such as medication use, age, and the multiple subtypes of MDD [28]. In this context, we believe that studies of a single symptom phenotype, for instance, anhedonia in MDD might help reduce the heterogeneity and find more reliable experimental results.

Meanwhile, it has been revealed that multiple and functionally specialized subfields of the hippocampus have their own signatures [29]. Continuing advances in structural magnetic resonance imaging (MRI) techniques, such as the automated hippocampal segmentation approach developed by Iglesias et al. [30], have made it possible to label hippocampal subareas and provide the volume information of each subarea automatically with subfield identification validity and reliability using ex vivo and ultrahigh-resolution MRI. Some studies have found hippocampal subfield-level volume reductions in MDD patients [23, 31], and atrophy of the hippocampal substructure may have the potential to represent a marker for depressive illness [32], rather than atrophy of the whole hippocampus. Thus, the aim of the present study was to investigate the volumetric alterations in the hippocampus as well as its subfields in MDD patients with and without anhedonia.

## Methods

### Participants

In the present study, a total of 70 MDD patients, including 40 MDD patients with anhedonia and 30 MDD patients without anhedonia, were recruited from the Department of Psychiatry, The First Affiliated Hospital, Zhejiang University School of Medicine. The inclusion criteria for MDD patients included the following: (1) aged from 18 to 45; (2) met the DSM-IV criteria for current unipolar MDD episode, which was assessed using Structured Clinical Interview for DSM-IV (SCID); (3) drug-naïve patients or recurrent depression with continued withdrawal for more than 3 months; (4) total score of 17-item Hamilton Depression Scale (HAMD-17) ≥ 17; (5) right-handedness; and (6) could follow the instructions to remain still during MRI scanning. For assignment to the MDD with anhedonia group, MDD patients must have been experiencing anhedonia according to Item 2 (loss of interest or pleasure) of the symptom criteria (A) for MDD in the DSM-IV and the threshold of the transformed score of the Snaith-Hamilton Pleasure Scale (SHAPS). Meanwhile, a total of 43 sex- and age-matched healthy controls (HCs) were recruited from local residents, hospital staff and students. All HCs were thoroughly interviewed and were free from any current or lifetime history of psychiatric disorders according to the DSM-IV criteria. The general exclusion criteria were as follows: (1) existence of any major medical disease, including cardiovascular, respiratory, endocrine and neurological diseases (e.g., epilepsy, brain trauma and stroke); (2) current use of any medication that might affect the central nervous system; (3) drug or alcohol dependence or abuse; (4) female with pregnancy; (5) with histories of psychotherapy and physical therapy, such as transcranial direct current stimulation (tDCS), transcranial magnetic stimulation (TMS), and electroconvulsive therapy (ECT); and (6) contraindications to MRI scan, including retractors or braces, metallic implants, and claustrophobia. The present study is one of our serial investigations focusing on MDD, and the recruitment of participants has been described in our previous studies [33–35]. This study was approved by the local Medical Ethics Committee of The First Affiliated Hospital, Zhejiang University School of Medicine. Each participant provided written informed consent prior to commencement of the study.

### Clinical assessment

The demographic and clinical data were collected by a self-designed questionnaire from all the participants. The Structured Clinical Interview for DSM-IV (SCID) was used for the diagnostic assessment of MDD and further psychiatric disorders, which was also administered to each subject. The HAMD-17, one of the most common clinician-rated scales in MDD, was used to assess the severity of depressive episodes [36]. The Chinese version of the SHAPS [37, 38], considered a reliable and valid self-report questionnaire, was used to evaluate the state of severe anhedonia in the study. The scales include 14 items and cover four domains (interests and pastimes, social interactions, sensory experiences and diet). Possible responses for each item include: strongly disagree, disagree, agree and strongly agree, with 1 for strongly agree and 4 for strongly disagree in raw scores. Considering that the raw SHAPS scores could not distinguish MDD patients with and without anhedonia, we used the binarized SHAPS score to evaluate the state of severe anhedonia. Namely, “agree” or “strongly agree” was recorded as a score of 0, while “disagree” or “strongly disagree” was recorded as 1. The total score after conversion to > 5 could distinguish the presence or absence of severe anhedonia, which was used in some previous studies [35, 39]. In this study, we defined severe anhedonia as a dichotomous variable with a SHAPS score > 5 and calculated the raw total scores of the SHAPS scale for MDD patients to assess the severity of anhedonia.

### MRI data acquisition

Imaging data were collected from all participants using a 3.0-T scanner (Signa, HDxt, GE healthcare, USA) with a standard birdcage head coil in the Magnetic Resonance Center at the First Affiliated Hospital of Zhejiang University School of Medicine. Each subject read the notes carefully prior to beginning the protocol and kept laying still with their eyes opened during the MRI experiment. The 3D T1-weighted structural image in the study was obtained by brain volume (BRAVO) sequence, and the parameters were chosen as follows: TR = Minimum (7.3 ms), TE = Minimum (3.0 ms), TI = 1100 ms, flip angle = 7, FOV = 256 * 256 mm^2^, Matrix = 256 * 256, slice thickness = 1 mm, bandwidth = 31.25 kHz, NEX = 1, slices = 192.

### Preprocessing and segmentation

Hippocampal subfield volumetric segmentation was used by the new FreeSurfer software (v. 6.0) algorithm [30] (Laboratory for Computational Neuroimaging, Athinoula A. Martinos Center for Biomedical Imaging, Charlestown, MA, USA; http://surfer.nmr.mgh.harvard.edu), which is based on the ex vivo MRI data that have achieved the automated segmentation of hippocampal subregions and have been proven to substantially increase the segmentation accuracy, especially the GCL within the dentate gyrus, the molecular layer (ML) within the subiculum and the CA subfields [40, 41]. The specific processing technology details of FreeSurfer have been explained in previous literature [42, 43]. Nevertheless, nineteen hippocampal subfield volumes, including CA1 (head and body), CA2/3 (head and body), CA4 (head and body), fimbria, granule cell and molecular layer of the dentate gyrus (GC-ML-DG, head and body), hippocampal–amygdaloid transition area (HATA), fissure, tail, molecular layer (ML, head and body), parasubiculum (Para), presubiculum (Pre, head and body), and subiculum (Sub, head and body), and three calculated region volumes (the whole hippocampus, head and body) were included within the study.

### Statistical analysis

Statistical analyses of demographic and clinical data were performed using the Social Sciences (SPSS) (version 26.0, SPSS Inc., Chicago, IL, USA). Categorical variable results are expressed as percentages (m/n), and continuous variable data are represented as the mean (standard deviation) for statistical description. The difference among the three diagnostic groups was performed by Chi-square tests (χ^2^) for categorical variables, while one-way analysis of variance (ANOVA, *post hoc* Bonferroni) was performed for continuous variables. Considering the confounders of age, sex, education years, and estimated intracranial volumes (eTIV), we performed the analysis of covariance (ANCOVA) for the volumetric differences in hippocampal subfields among the three groups controlling for age, sex, education years, and eTIV in further analyses. Post hoc tests were calculated for intergroup comparisons. The partial correlation between the clinical data and radiographic indices was analysed with sex, age, education years, and eTIV were used as covariables. The Benjamini-Hochberg method (BH) was used to adjust the *p* values, and a two-sided *p* < 0.05 significance level was considered significant in the study.

## Results

### Demographic and clinical characteristics

There were one hundred thirteen subjects, including thirty MDD patients with severe anhedonia, forty MDD patients without anhedonia and forty-three healthy controls, in the study. All demographic and clinical information of the three groups is presented in Table 1. There were no significant differences in age, sex or education years among all three groups. Meanwhile, illness duration and HAMD-17 scores showed no statistically significant difference between MDD patients with severe anhedonia and MDD patients without anhedonia.

**Table 1:** Demographic and clinical characteristics for all subjects (n = 113)

	MDD with anhedonia, n = 30 means (SD)	MDD without anhedonia, n = 40 means (SD)	HCs, n = 43 means (SD)	Analysis *F/χ* ^*2*^	*p*-values
Age (years)	27.86(7.20)	31.25(7.27)	28.25(8.17)	2.242	0.111
Gender (Male/Female)	7/23	10/30	17/26	2.967	0.112
Education years	13.86(3.00)	14.36(2.83)	15.00(2.27)	1.631	0.201
Illness duration (months)	22.10(21.58)	22.76(27.74)		0.273	0.915
SHAPS score	45.53(5.23)^***a***^	35.00(5.13)^***b***^		0.011	0.000
HAMD score	25.73(3.30)^***c***^	24.13(3.63)^***d***^		0.513	0.061

HAMD, Hamilton Depression Scale; HCs, Healthy Controls; MDD, Major Depressive Disorder; SD, Standard Deviation; SHAPS, Snaith-Hamilton Pleasure Scale

SHAPS score ranges: *a*, 34–60; *b*, 20–42

HAMD score ranges: *c*, 19–36; *d*, 18–34

### Hippocampal subfield analysis

**Fig. 1:**
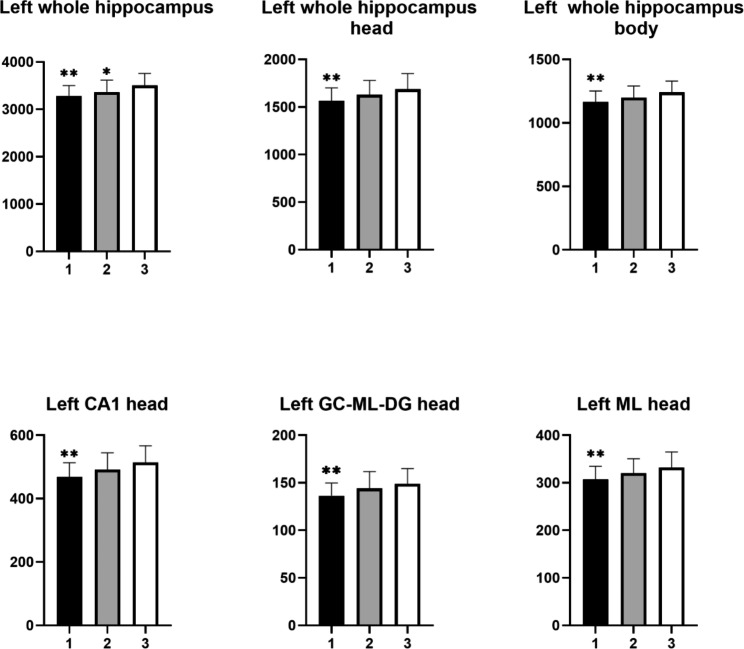
Comparisons of hippocampal subfield volumes between the three groups. Asterisk represents significantly different volume versus HC group after Bonferroni correction. ** *p* < 0.01; *p < 0.05. Error bar represents one standard error. 1, MDD with anhedonia; 2, MDD without anhedonia; 3, Healthy Controls. CA, cornu ammonis; GC-ML-DG, Granule cell and molecular layer of the dentate gyrus; ML, molecular layer

Table 2 presents the volumes of the total hippocampus and its subfields including CA1, CA2/3, CA4, fimbria, GC-ML-DG, HATA, fissure, tail, ML, Para, Pre, and Sub. Among them, CA1-CA4, GC-ML-DG, ML, and Pre were divided into the head and body as well as the whole hippocampus. ANOVA showed that the significant differences were concentrated in the left hippocampus among the three groups. Significant differences were observed in the volumes of the hippocampal head, mainly in the CA1 head, GC-ML-DG head and ML head. After a post hoc analysis, we found that although the whole left hippocampus was significantly smaller in both groups of patients with MDD, only MDD patients with anhedonia displayed significant volume reductions in the left hippocampus head, body, CA1 head, GC-ML-DG head and ML head when comparing to HCs (Fig. 1). No significant difference was found in the volumes of the hippocampal subfields between MDD patients without anhedonia and HCs, either the two groups of MDD patients.

**Table 2:** Volume of gray matter in n hippocampus subregion for all subjects (n = 113)

	MDD with anhedonia, n = 30 means (SD)	MDD without anhedonia, n = 40 means (SD)	HCs, n = 43 means (SD)	*F*	*p*-values	Adjusted *p*-values
**Left hemisphere**						
Whole hippocampus	3284.76(217.83)	3364.32(249.98)	3505.45(254.54)	7.797	< 0.001	**0.028**
Whole hippocampal head	1564.86(136.56)	1629.62(149.27)	1686.95(164.63)	5.714	0.004	**0.038**
Whole hippocampal body	1166.16(85.34)	1199.06(91.96)	1241.56(88.57)	6.563	0.002	**0.030**
Whole hippocampal tail	553.74(61.78)	535.64(98.25)	576.95(59.21)	3.086	0.050	0.146
CA1 head	468.54(44.87)	491.69(53.29)	514.02(52.84)	7.083	0.001	**0.028**
CA1 body	109.89(21.41)	114.67(24.98)	118.04(20.45)	1.172	0.314	0.406
CA3 head	104.61(12.94)	109.09(16.38)	112.65(13.64)	2.723	0.070	0.162
CA3 body	80.09(14.15)	80.67(13.44)	84.71(12.85)	1.380	0.256	0.352
CA4 head	113.50(10.27)	119.42(14.90)	123.00(13.11)	4.640	0.012	0.064
CA4 body	116.17(10.03)	117.60(9.75)	122.89(10.30)	4.779	0.010	0.064
GC-ML-DG head	136.48(13.20)	144.05(17.69)	148.83(16.11)	5.276	0.006	**0.048**
GC-ML-DG body	131.10(10.77)	133.28(10.25)	138.25(11.90)	4.148	0.018	0.081
ML head	307.40(27.45)	320.35(30.16)	332.28(32.67)	5.930	0.004	**0.038**
ML body	218.56(20.59)	224.81(22.66)	233.27(18.81)	4.632	0.012	0.057
Pre head	134.52(14.08)	135.22(13.57)	140.52(18.47)	1.700	0.187	0.284
Pre body	172.63(24.15)	179.67(32.62)	185.28(20.91)	2.027	0.137	0.251
Sub head	251.97(25.00)	257.30(25.04)	266.95(23.49)	3.577	0.031	0.115
Sub body	190.15(24.72)	197.18(23.42)	201.34(25.20)	1.854	0.161	0.263
Para	56.57(8.73)	56.97(9.69)	57.71(12.63)	0.109	0.897	0.897
Fissure	136.41(18.61)	147.17(26.45)	145.52(27.43)	1.770	0.175	0.275
HATA	53.10(6.85)	55.64(8.02)	56.61(8.07)	1.856	0.161	0.263
Fimbria	85.74(18.29)	91.06(22.82)	92.17(17.26)	1.022	0.363	0.432
**Right hemisphere**						
Whole hippocampus	3453.86(274.02)	3521.48(340.66)	3620.17(275.03)	2.853	0.062	0.160
Whole hippocampal head	1677.25(168.83)	1714.65(202.06)	1760.23(171.19)	1.885	0.157	0.263
Whole hippocampal body	1197.59(100.92)	1220.07(116.70)	1256.86(88.33)	3.149	0.047	0.146
Whole hippocampal tail	579.01(67.81)	586.77(66.13)	603.08(63.64)	1.311	0.274	0.365
CA1 head	515.83(61.25)	536.49(74.19)	548.66(57.73)	2.266	0.109	0.217
CA1 body	126.97(22.76)	131.55(26.35)	130.38(18.73)	0.364	0.696	0.785
CA3 head	117.21(17.13)	118.92(19.02)	121.38(17.51)	0.499	0.609	0.705
CA3 body	93.44(14.96)	94.37(14.23)	95.39(14.49)	0.161	0.851	0.892
CA4 head	124.75(13.42)	126.41(17.50)	129.91(15.44)	1.050	0.353	0.432
CA4 body	117.87(10.97)	121.33(11.99)	124.12(11.90)	2.536	0.084	0.184
GC-ML-DG head	150.39(17.32)	153.39(22.02)	158.15(18.46)	1.481	0.232	0.329
GC-ML-DG body	131.29(12.07)	135.97(14.03)	139.74(11.42)	3.998	0.021	0.084
ML head	328.17(34.79)	337.28(38.90)	345.66(34.10)	2.094	0.128	0.245
ML body	231.03(24.23)	235.73(22.77)	243.11(20.09)	2.770	0.067	0.162
Pre head	133.14(15.61)	132.53(13.39)	137.80(14.79)	1.594	0.208	0.305
Pre body	161.20(18.73)	162.45(25.33)	171.25(20.04)	2.483	0.088	0.185
Sub head	196.28(31.45)	200.91(32.10)	201.85(27.14)	0.329	0.721	0.793
Sub body	252.18(25.39)	254.50(30.56)	267.28(26.60)	3.336	0.039	0.133
Para	53.65(9.75)	52.14(13.83)	55.63(9.09)	1.025	0.362	0.432
Fimbria	83.61(16.82)	84.17(21.43)	85.60(16.97)	0.114	0.892	0.897
HATA	57.83(9.40)	56.58(7.68)	61.19(9.41)	2.998	0.054	0.148
Fissure	156.21(22.03)	160.11(26.98)	157.31(28.02)	0.215	0.807	0.866

CA, Cornu ammonis; GC-ML-DG, Granule cell and molecular layer of the dentate gyrus; HATA, Hippocampal–amygdaloid transition area; HCs, Healthy Controls;

MDD, Major Depressive Disorder; ML, Molecular layer; Para, Parasubiculum; Pre, Presubiculum; SD, Standard Deviation; Sub, Subiculum

Moreover, to eliminate the effect of potential confounders such as age, sex, education years, and eTIV, we conducted a series of covariance analyses and found significant volumetric differences among the three groups in the left whole hippocampus (*F* = 5.036, *p* = 0.008), left whole hippocampal body (*F* = 3.930, *p* = 0.023), left CA1 head (*F* = 4.041, *p* = 0.020), left CA4 body (*F* = 4.425, *p* = 0.014) and left GC-ML-DG body (*F* = 3.153, *p* = 0.047). However, none were significant after *BH* correction.

However, no association between hippocampal subfield volumes and clinical characteristics was found in either the subset of patients with anhedonia or in the patient group as a whole.

## Discussion

In this study, the volumetric alterations of the hippocampus as well as its subfields in MDD patients with and without anhedonia were investigated. It was found that volume was reduced in the whole left hippocampus in MDD patients, including both the anhedonic group and the nonanhedonic group, when compared with HCs. Moreover, when comparing the two MDD subgroups with HCs, we found significantly smaller volumes in the left hippocampus head, body, CA1 head, DG head and ML head in MDD patients with anhedonia; however, this difference was not detected in MDD patients without anhedonia, which might reflect characteristics of the hippocampal structures in MDD patients with anhedonia.

In the present study, significant differences in hippocampal volumes were predominantly in the left hemisphere, and both MDD subgroups (irrespective of the anhedonic subset) showed a significantly decreased volume in the whole left hippocampus. It is acknowledged that the two brain hemispheres are asymmetric in anatomy and function. The left hemisphere is considered to be involved in the pursuit of pleasure, as well as in the enjoyment when a reward is attained [44]. Consistent with our findings, previous imaging studies reported that MDD patients exhibited a significantly smaller volume of the left hippocampus [31, 45], which confirmed the asymmetry of hippocampal structures in MDD.

More importantly, due to the automatic method that was used to analyse the hippocampal subfields, we found that MDD patients with anhedonia (not those without anhedonia) exhibited smaller volumes in the hippocampal head, especially in the CA1, DG and ML, than HCs. Functional segregation was observed in the hippocampus along the longitudinal axis, and the anterior hippocampus seemed to be closely associated with emotional processing, while the posterior hippocampus was more associated with cognitive functions [46, 47]. Prior studies also reported more severe atrophy in the hippocampal head in MDD patients [48]. Moreover, the hippocampal head, as a characteristic of digitations in the hippocampus, is susceptible to damage with higher excitatory cell density and lower inhibitory cell density [49, 50]. A study indicated that traumatic brain injury-induced hippocampal damage mainly involved the hippocampal head, which suggested a more severe neuronal loss in the anterior hippocampus [51].

Meanwhile, as the major component of the hippocampal head, the anterior CA1 subfield, as well as the DG and ML, showed significant atrophy in MDD patients with anhedonia in this study, which might be associated with the deficits in reward processing. The CA1 subfield is the largest area in the hippocampus and is composed of different layers [52]. Previous studies have suggested the CA1 place cells are related to the accumulation of place fields near learned rewarded locations and have the power to encode rewards [53, 54]. Meanwhile, the activities of DA in CA1 are important in hippocampal-dependent reward learning [55], and the DA inputs from VTA to the CA1 subfield have large effects on spatial memory and are involved in the reward learning modulation [56]. A recent study reported that the anhedonic behavior was associated with dendritic spine elongation in the CA1 subregion of the hippocampus [57], which suggested that abnormalities in the structure and function of the CA1 subfield contribute to anhedonia.

The DG region is largely composed of granule cells and can receive multiple sensory inputs from the perirhinal and lateral entorhinal cortex [58]. The molecular layer stretches as a dark band from the DG along the CA subfields to the subiculum [59]. It consists of interneuron synapses and contains the dendrites from DG neurons [60], and it is speculated that a lower volume of the molecular layer could reflect the loss of dendritic connections or DG neurons [59]. The DG is likely to participate in the natural reward-associated memory processing and has an important influence on memory encoding, reward memory formation and recall [61, 62].

Numerous studies have indicated that CA1 and DG subfield atrophy is related to the putative neurobiological mediation pathways, including the brain-derived neurotrophic factor (BDNF) genotype [63, 64], oxidative stress [65, 66] and hypothalamic-pituitary-adrenal (HPA) axis dysregulation [67, 68], which are closely linked to the pathogenesis of anhedonia in MDD. Recent studies have reported that MDD patients with anhedonia exhibit distinct alterations in HPA axis activity [69], overactivation of the inflammation [70], and hypermetabolism of BDNF [71], indicating the important effect of these factors in anhedonia. Moreover, in MDD individuals, it was found that the smaller volume in CA1 [72, 73] and the functional connectivity alterations of CA1 [74] were related to history of childhood trauma, which is a strong predictor for anhedonic depression [75, 76]. These overlapping findings support our results and reveal the possible interrelationship between the CA1 and DG, ML atrophy and anhedonia in MDD.

## Limitations

Certain limitations should be mentioned. First, this study included a relatively small sample of each group, and we only recruited patients with moderate to severe major depressive disorder to minimize the clinical heterogeneity, which might restrict the generalization of our findings. Second, we did not distinguish the different components of anhedonia due to the modest sample size, and thus, we were unable to investigate the effects of different anhedonia subtypes on structural alterations of the hippocampus in MDD patients. Meanwhile, because the effects of confounding factors such as age, sex, education years, and eTIV were not taken into account during ANOVA, the results in our study should be viewed as preliminary, and larger cohorts on the anhedonic subtype of depression are required in the future. Finally, this study was cross-sectional in nature and cannot explain the direct causal relationship between altered hippocampal structures and anhedonia in MDD patients.

## Conclusion

These preliminary findings suggest that MDD patients with anhedonia exhibit unique atrophy of the hippocampus and that subfield abnormalities in the left CA1 and DG might be associated with anhedonia in MDD.

## Data Availability

The datasets generated and/or analyzed during the current study are not publicly available due to privacy and ethical restrictions but are available from the corresponding author on reasonable request.
